# Static vs. cidal: it’s not complex; it’s simply incorrect

**DOI:** 10.1128/aac.00513-25

**Published:** 2025-07-21

**Authors:** Brad Spellberg, Noah Wald-Dickler, Paul Holtom, Pascal Meyer-Sautter, Austin Camp, Alejandro Diaz Diaz, Ranya Buhamad, Ali Sebastian Meza Vazquez, Gloria Mayela Aguirre-Garcia, Matthew Stanton, Susan M. Butler-Wu, Isabelle Chiu, Zeynep Ergenc, Guru Bhoojhawon, Rita Murri, Alberto Enrico Maraolo, Gabriela Cabanilla, Niccolò Riccardi, Vhudzani Tshisevhe, Curtis Behenna, Karen S. Williams, Wesley D. Kufel, Natalia Wojciaczyk, Bernardo Vidal Pimentel, Ahmed Muyidi, Rodrigo P.L. Costa, Fabrizio Motta, Émilie Bortolussi-Courval, Todd C. Lee, Emily McDonald, Bassam Ghanem, Zachary Nelson

**Affiliations:** 1 Los Angeles General Medical Center 23336 , Los Angeles, California, USA; 2 Department of Internal Medicine, Kantonsspital Glarus 30610 , Glarus, Switzerland; 3 Union University College of Pharmacy, Jackson, Tennessee, USA; 4 Department of Pediatric Infectious Diseases, Hospital Pablo Tobón Uribe, Hospital General de Medellín 576742 , Medellín, Colombia; 5 Jaber Al-Ahmad Al-Sabah Hospital, Ministry of Health, Kuwait, Kuwait; 6 Department of Internal Medicine, Mexican Institute of Social Security, Western National Medical Center, High Specialty Medical Unit 541025 , Guadalajara, Mexico; 7 Tecnologico de Monterrey, Escuela de Medicina y Ciencias de la Salud 70656, Monterrey, Mexico; 8 Froedtert and the Medical College of Wisconsin, Medical College of Wisconsin School of Pharmacy 492267, Milwaukee, Wisconsin, USA; 9 Division of Infectious Diseases, University of Alberta, Edmonton, Alberta, Canada; 10 Vaccine Institute, City St. George’s University of London 3163, London, United Kingdom; 11 Paediatric Infectious Diseases and Immunology, Great Ormond Street Hospital for Children 4956 , London, United Kingdom; 12 Division of Pediatric Hospital Medicine, Driscoll Children’s Hospital, Corpus Christi, Texas, USA; 13 Infectious Diseases Unit, Fondazione Policlinico Gemelli IRCCS, Rome, Italy; 14 Section of Infectious Diseases, Department of Clinical Medicine and Surgery, University of Naples Federico II 9307, , Naples, Italy; 15 Department of Pharmacy, Division of Infectious Diseases, Department of Internal Medicine, The University of New Mexico Health Sciences Center 12289, Albuquerque, New Mexico, USA; 16 TB Reference Centre and Laboratory, ASST Grande Ospedale Metropolitano Niguarda 9338, Milan, Italy; 17 Lancet Laboratories 518386, Rustenburg, South Africa; 18 Department of Medical Microbiology, University of Pretoria 574011, Pretoria, South Africa; 19 Department of Infectious Disease, Ascension St John Medical Center 159297 , Tulsa, Oklahoma, USA; 20 Department of Pharmacy, Guthrie Robert Packer Hospital 23128, Sayre, Pennsylvania, USA; 21 State University of New York at Binghamton School of Pharmacy and Pharmaceutical Sciences 14787, Binghamton, New York, USA; 22 State University of New York Upstate Medical University, Syracuse, New York, USA; 23 The Guthrie Clinic, Sayre, Pennsylvania, USA; 24 Department of Internal Medicine, Hospital da Luz Lisboa 199396 , Lisbon, Portugal; 25 King Faisal Medical City, Riyadh, Saudi Arabia; 26 Hospital Mater Dei Salvador 223018, Salvador, Brazil; 27 Hospital Cárdio Pulmonar da Bahia 534569 , Salvador, Brazil; 28 Division of Infectious Diseases, Santa Casa de Porto Alegre, Porto Alegre, Brazil; 29 Faculté de Médecine et des Sciences de la Santé, Université de Sherbrooke 7321, Sherbrooke, Canada; 30 Division of Infectious Diseases, Department of Medicine, McGill University 5620, Montreal, Canada; 31 Division of General Internal Medicine, Research Institute of the McGill University Health Centre 507266, Montreal, Canada; 32 Pharmaceutical Care Department, King Abdulaziz Medical City, National Guard Health Affairs 48022, Jeddah, Saudi Arabia; 33 HealthPartners and Park Nicollet Health Services, Minneapolis, Minnesota, USA; Columbia University Irving Medical Center, New York, New York, USA

**Keywords:** cidal, static, antibiotic

## LETTER

In the first sentence of their recent study (1), Gil-Gil and Berryhill cite a 2018 review article (2) to support the statement, “The clinical outcomes of antibiotic treatments involving the administration of bacteriostatic (which inhibit bacterial growth) or bactericidal (which kill bacteria) antibiotics are complex and context-dependent.”

However, the concept of static/cidal antibiotics is neither complex nor context-dependent; it is rather a myth. Regrettably, this dogma continues to be raised in modern literature despite two fundamental flaws. First, randomized controlled trials (RCTs) have failed to establish any relationship between static or cidal antibiotics and clinical efficacy (2). The cited review article summarizes the results of 56 RCTs directly comparing the efficacy of static vs cidal antibiotics across a variety of diseases (2). Four more such RCTs were subsequently published in 2019 (3–6). Overall, 53 (88.3%) RCTs found no difference in efficacy, six (10%) found the static antibiotic, linezolid, to be superior in efficacy to cidal antibiotics, and only one (1.7%) found a static antibiotic, tigecycline, to be inferior in efficacy to a cidal antibiotic. In the tigecycline trial, standard dosing (50 mg twice daily) resulted in suboptimal drug target level attainment for ventilator-associated pneumonia (VAP), and a subsequent RCT in which the dose of tigecycline was doubled achieved non-inferiority to cidal therapy for VAP (7). Other, more recent meta-analyses have reinforced that there is no discernible benefit from cidal antibiotics over their static counterparts (8, 9). Thus, static and cidal antibiotics do not intrinsically differ in clinical efficacy.

The second flaw is the myth that the word “bacteriostatic” means “inhibit bacterial growth,” whereas “bactericidal” means “kill bacteria.” As previously discussed (2, 9), the microbiological definition of “static” is that the minimum bactericidal concentration (MBC) of the antibiotic is more than fourfold greater than its minimum inhibitory concentration (MIC). In contrast, a “cidal” antibiotic is one for which the MBC is not more than fourfold above the MIC. MBC is defined as the concentration at which a 1,000-fold reduction in bacterial density is achieved at 24 hours of culture under specific conditions. MIC is the concentration that inhibits visible growth. Why there should be any importance or clinical impact of the relationship between MBC and MIC has never been described. Moreover, some antibiotics (e.g., chloramphenicol and clindamycin) can be either static or cidal depending on experimental conditions (2, 10).

Thus, the static and cidal concepts are unrelated to inhibiting growth vs killing bacteria. Indeed, every single “static” antibiotic on the market today kills bacteria—there are no antibiotics that inhibit growth but do not kill. This dogma must stop being taught to clinicians and be put out to pasture as a relic of a bygone era in scientific literature.

The terms static and cidal are misleading, have no demonstrated clinical relevance, may lead to suboptimal antimicrobial selection (or toxicity), and should be abandoned. We implore authors, peer reviewers, and journal editors to stop perpetuating the concept of static and cidal antibiotics, given the aforementioned evidence demonstrating it is a myth. In a clinical context, these terms are not complex. Rather, they are simply incorrect.
